# Comparison between postcontrast thin-slice T1-weighted 2D spin echo and 3D T1-weighted SPACE sequences in the detection of brain metastases at 1.5 and 3 T

**DOI:** 10.1186/s13244-024-01643-6

**Published:** 2024-03-14

**Authors:** Josef Vymazal, Zuzana Ryznarova, Aaron M. Rulseh

**Affiliations:** Department of Radiology, Na Homolce Hospital, Roentgenova 2, Prague, 150 30 Czech Republic

**Keywords:** Brain metastasis, Imaging sequences, Magnetic resonance imaging, Radiosurgery

## Abstract

**Objectives:**

Accurate detection of metastatic brain lesions (MBL) is critical due to advances in radiosurgery. We compared the results of three readers in detecting MBL using T1-weighted 2D spin echo (SE) and sampling perfection with application-optimized contrasts using different flip angle evolution (SPACE) sequences with whole-brain coverage at both 1.5 T and 3 T.

**Methods:**

Fifty-six patients evaluated for MBL were included and underwent a standard protocol (1.5 T, *n* = 37; 3 T, *n* = 19), including postcontrast T1-weighted SE and SPACE. The rating was performed by three raters in two sessions > six weeks apart. The true number of MBL was determined using all available imaging including follow-up. Intraclass correlations for intra-rater and inter-rater agreement were calculated. Signal intensity ratios (SIR; enhancing lesion, white matter) were determined on a subset of 46 MBL > 4 mm. A paired *t*-test was used to evaluate postcontrast sequence order and SIR. Reader accuracy was evaluated by the coefficient of determination.

**Results:**

A total of 135 MBL were identified (mean/subject 2.41, SD 6.4). The intra-rater agreement was excellent for all 3 raters (ICC = 0.97–0.992), as was the inter-rater agreement (ICC = 0.995 SE, 0.99 SPACE). Subjective qualitative ratings were lower for SE images; however, signal intensity ratios were higher in SE sequences. Accuracy was high in all readers for both SE (*R*^2^ 0.95–0.96) and SPACE (*R*^2^ 0.91–0.96) sequences.

**Conclusions:**

Although SE sequences are superior to gradient echo sequences in the detection of small MBL, they have long acquisition times and frequent artifacts. We show that T1-weighted SPACE is not inferior to standard thin-slice SE sequences in the detection of MBL at both imaging fields.

**Critical relevance statement:**

Our results show the suitability of 3D T1-weighted turbo spin echo (TSE) sequences (SPACE, CUBE, VISTA) in the detection of brain metastases at both 1.5 T and 3 T.

**Key points:**

• Accurate detection of brain metastases is critical due to advances in radiosurgery.

• T1-weighted SE sequences are superior to gradient echo in detecting small metastases.

• T1-weighted 3D-TSE sequences may achieve high resolution and relative insensitivity to artifacts.

• T1-weighted 3D-TSE sequences have been recommended in imaging brain metastases at 3 T.

• We found T1-weighted 3D-TSE equivalent to thin-slice SE at 1.5 T and 3 T.

**Graphical Abstract:**

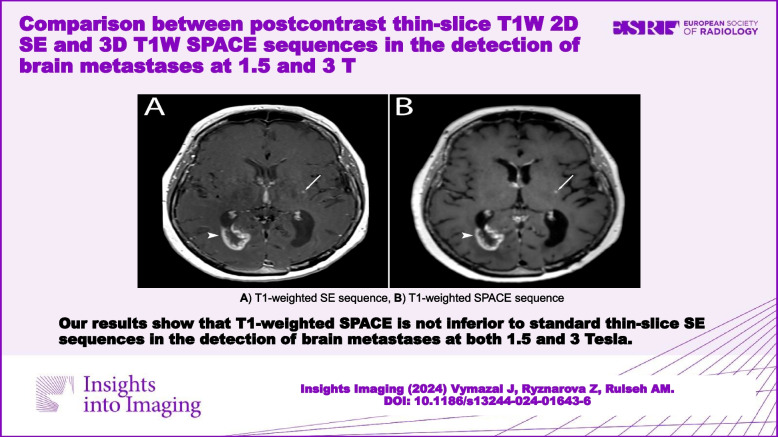

## Introduction

Magnetic resonance imaging (MRI) is superior to computer-assisted tomography (CT) in the detection of brain metastases and distinguishing them from similar-appearing lesions [1]. MRI is also routinely used in the workup of and treatment planning for affected patients [2]. Historically, patients with numerous metastatic brain lesions were often treated by whole-brain radiotherapy (WBR), and thus, the exact number of lesions present was more of an academic question. However, with the emergence and further development of targeted radiosurgery techniques such as Leksell Gamma Knife or CyberKnife therapy, and a trend away from WBR, there is a greater need to accurately detect small and multiple metastatic brain lesions.

In daily practice, there is no guarantee that all brain metastases will be reliably detected on postcontrast MRI, with small metastatic lesions being particularly problematic. The diagnostic yield of MRI in the detection of brain metastases depends on a number of factors, particularly the type of postcontrast T1-weighted sequence and its acquisition properties (e.g., spatial resolution), as well as gadolinium-based intravenous contrast relaxivity, dose and timing with respect to administration, and postcontrast sequence acquisition [3–6]. In general, postcontrast T1-weighted spin echo (SE) sequences have been shown to be superior to postcontrast T1-weighted gradient echo sequences in the detection of small metastatic brain lesions [7–10], although gradient echo sequences are applied and recommended in many centers [11, 12]. Postcontrast T1-weighted SE sequences with a large slice thickness (approximately 6 mm) have however been shown to be inferior to 3D gradient echo T1-weighted sequences, especially in the detection of small metastatic lesions [13]. Thin-slice 2D SE sequences may enable the detection of even small metastases; however, the disadvantages include imaging artifacts and long acquisition times if whole-brain coverage is desired [14].

Postcontrast 3D turbo spin echo (TSE) T1-weighted sequences (SPACE, CUBE, VISTA) may achieve very thin slice thicknesses (1 mm or less) and have been recommended for the detection of metastases at 3 T, but their utility at 1.5 T has not yet been well evaluated [12]. Thus, to further investigate proper sequence use, and to also evaluate the use of 3D T1-weighted TSE sequences at 1.5 T, we compared the accuracy of three readers in detecting metastatic brain lesions using very thin-slice T1-weighted 2D SE and T1-weighted SPACE sequences with whole-brain coverage, at both at 1.5- and 3-T field strengths.

## Methods

Fifty-six patients with suspected or known brain metastases were included in the study (29 males; mean age 63.4 years, range 19–87). Thirty-seven examinations were conducted at 1.5 T (Siemens Avanto, Erlangen, Germany) and 19 examinations at 3 T (Siemens Skyra, Erlangen, Germany). All subjects underwent a standard brain imaging protocol, including standard proton density-weighted, T2-weighted, and fluid-attenuated inversion recovery sequences, as well as standard diffusion-weighted imaging. All subjects were additionally imaged with a thin-slice T1-weighted 2D SE sequence (1.5 T: TR = 483–630 ms, TE = 17 ms, in-plane resolution = 0.94 × 0.94 mm, slice thickness = 3 mm, slice gap = 3.3 mm; 3 T: TR = 640–871 ms, TE = 8.2–10 ms, in-plane resolution = 0.43 × 0.43 mm, slice thickness = 2–3 mm, slice gap = 2.4–3.9 mm). Furthermore, a 3D T1-weighted sampling perfection with application-optimized contrasts using different flip angle evolution (SPACE) sequence was acquired in all subjects (1.5 T: TR = 500 ms, TE = 26 ms, resolution = 1 mm isotropic; 3 T: TR = 700 ms, TE = 11–12 ms, in-plane resolution = 0.75–0.91 mm, slice thickness = 0.9–1 mm). Seven of the SPACE sequences at 3 T had 0.9-mm isotropic resolution, while the remainder had slightly higher in-plane resolution with a slice thickness of 1 mm. The median acquisition time for the SE sequences was 10.4 min (range 8.3–16.8) at 1.5 T and 9.9 min (range 7.7–15.7) at 3 T, while the acquisition time of the SPACE sequences was a median of 4.6 min at 1.5 T and 4.3 min (range 3.9–5) at 3 T. The order of the T1-weighted sequences after contrast injection was varied, such that in 26 patients the SE sequence preceded the SPACE sequence, while in 30 patients the SPACE sequence preceded the SE sequence. Intravenous gadolinium-based contrast agents were administered at standard doses of 0.1 mL/kg. All patients received gadobutrol (Bayer HealthCare Pharmaceuticals, Berlin, Germany), with the exception of one patient who received gadoteridol (Bracco, Milan, Italy) and one patient who received gadobenate dimeglumine (Bracco, Milan, Italy). The study was approved by the ethics committee of Na Homolce Hospital, and all subjects provided signed, informed consent to participate in the study.

The rating was performed in 2 sessions at least 6 weeks apart by 3 raters (R1, R2, R3) with 11–29 years of experience (R1, 11 years; R2, 29 years; R3, 19 years). Images were evaluated using the Papaya image viewer (build 1455; https://rii-mango.github.io/Papaya), with the images presented in 3 orthogonal planes. The images were de-identified and the subject order was randomized for each session. The raters were instructed to count the number of likely metastatic lesions in the brain and to ignore enhancement outside the brain (e.g., meningeal enhancement), or any enhancement in the immediate area of any apparent intervention (e.g., resection or biopsy). The raters had access to only T1-weighted post-contrast images, either SE or SPACE, in each session, and each subject had only 1 sequence (SE or SPACE) in each session. Therefore, each rater evaluated 56 image volumes, covering 56 subjects, per session. The true number of metastatic lesions was determined after rating was finished, using all sequences available for that session, as well as radiological reports and follow-up imaging. The raters further provided subjective quality ratings (1–4) for each sequence, where 1 corresponded to “poor” and 4 to “excellent.”

Statistical analyses were performed using R version 3.6 [15]. The Mann–Whitney *U* test was employed to analyze potential differences in the number of metastatic lesions detected at 1.5 T versus 3 T. A paired *t*-test was used to evaluate the impact of sequence order with respect to contrast administration (first or second) on the number of metastatic lesions reported. Inter- and intra-rater metrics were determined by intraclass correlations (ICC), using a two-way model with the average of all raters as the basis. A mixed effects ICC model was used to determine intra-rater agreement with respect to the number of metastatic lesions detected in both sequences (SE versus SPACE for each rater). A random effects ICC model was used to evaluate inter-rater agreement in the number of metastatic lesions in each sequence (agreement in all raters for SE, agreement in all raters for SPACE). Finally, a mixed-effects ICC model was used to explore inter-rater agreement in qualitative ratings for sequence type (agreement in all raters for SE, agreement in all raters for SPACE). A paired *t*-test was further used to compare pooled quality ratings (all raters). The accuracy of ratings was evaluated by the coefficient of determination using the scikit-learn package in Python 3.8.

Signal intensity ratios were determined on a subset of 46 metastatic lesions (28 metastases at 1.5 T and 18 metastases at 3 T) that measured > 4 mm in at least two directions. Signal intensities were measured from the enhancing portion of lesions, with the frontal white matter [16] used as a reference region in the calculation of signal intensity ratios. A paired *t*-test was then used to assess differences between sequences (SE and SPACE) at 1.5 T and 3 T.

## Results

**Fig. 1:**
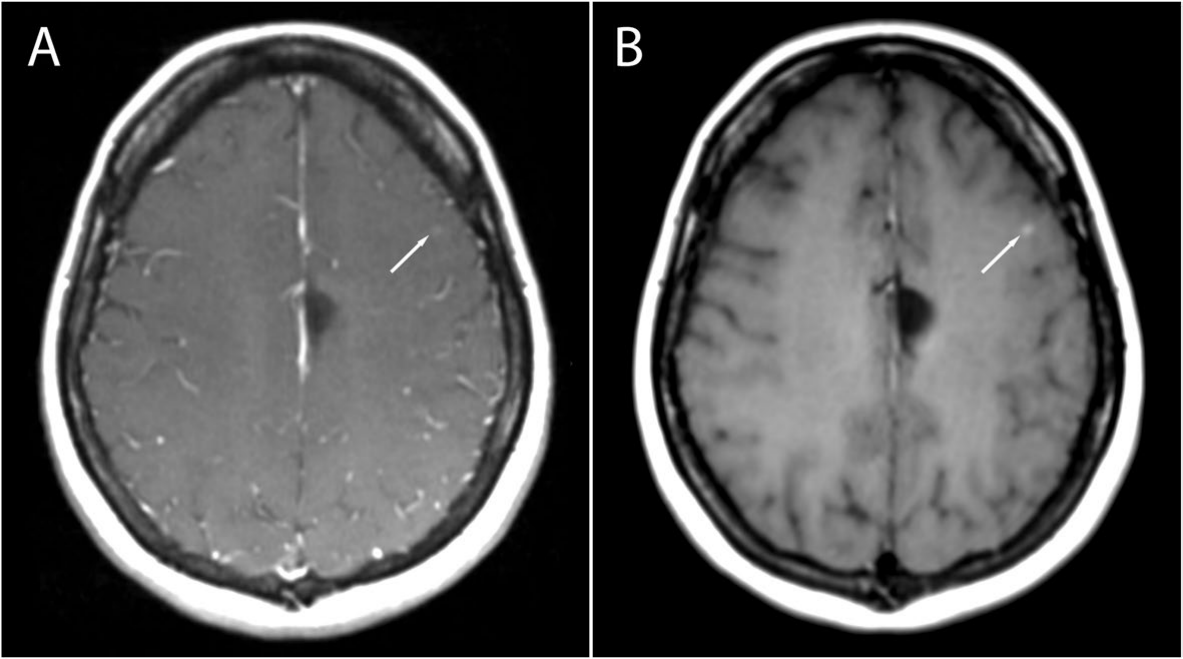
Small metastasis in the left frontal region in a 39-year-old female at 1.5 T, Gadovist 0.1 mL/kg. **A** T1-weighted SE sequence. **B** T1-weighted SPACE sequence. The SE sequence was acquired first

**Fig. 2:**
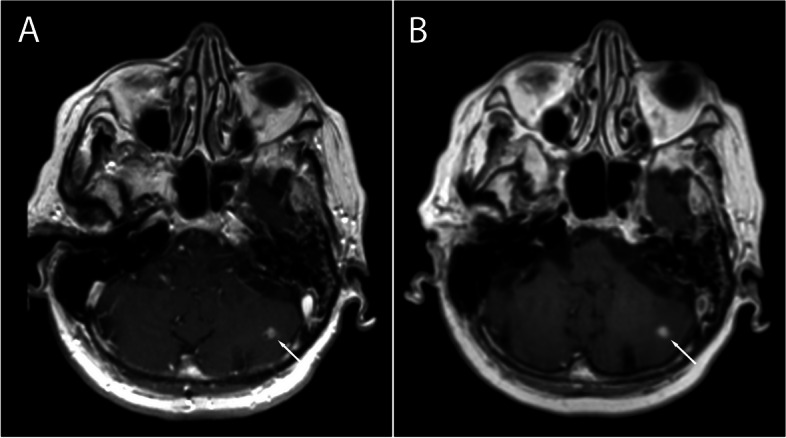
Small metastasis in the left cerebellar hemisphere in an 80-year-old female at 1.5 T, Gadovist 0.1 mL/kg. **A** T1-weighted SE sequence. **B** T1-weighted SPACE sequence. The SE sequence was acquired first

**Fig. 3:**
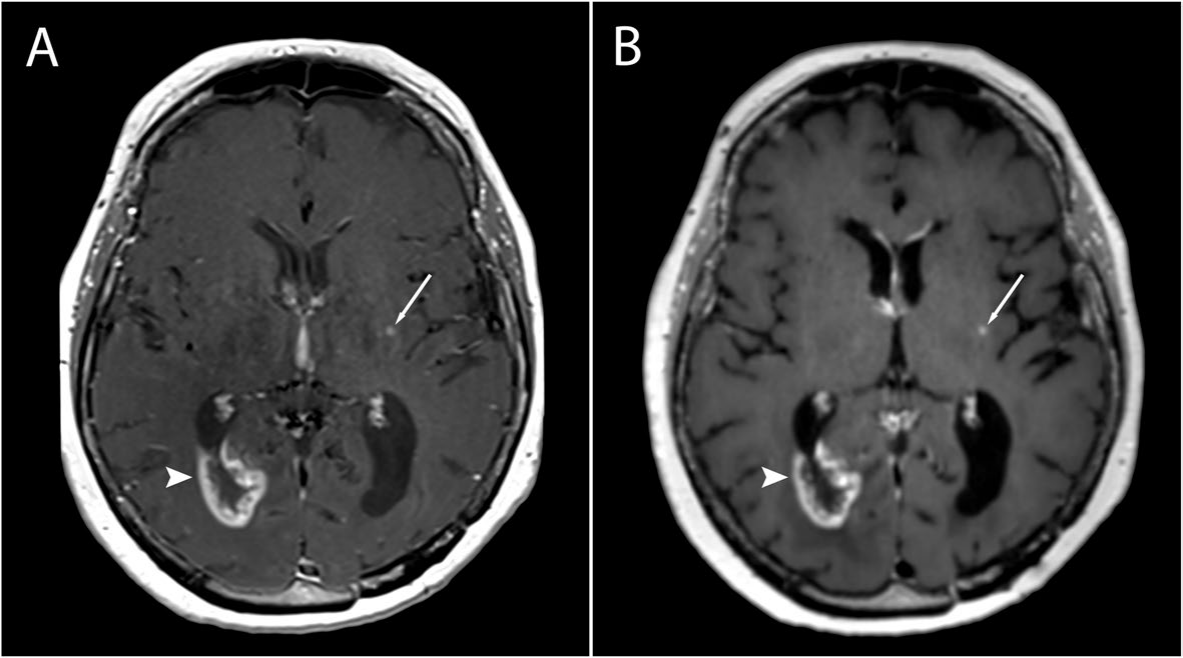
**A** T1-weighted SE sequence. **B** T1-weighted SPACE sequence. Metastases in the left basal ganglia (arrow) and right occipital region (arrowhead) in a 64-year-old female at 3 T, Gadovist 0.1 mL/kg. The SPACE sequence was acquired first

**Fig. 4:**
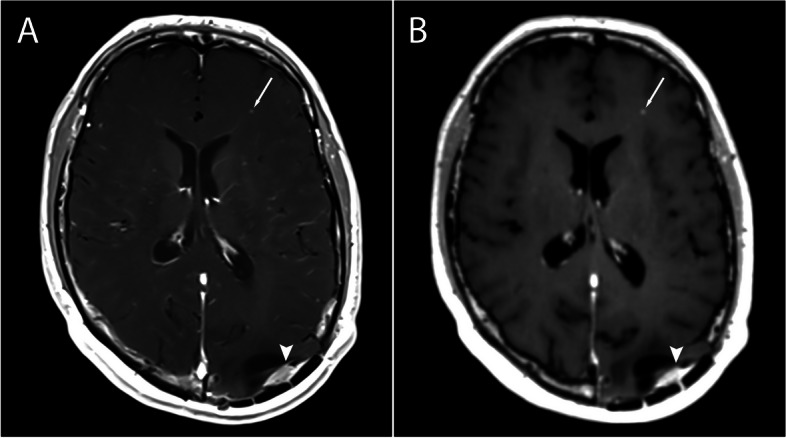
**A** T1-weighted SE sequence. **B** T1-weighted SPACE sequence. Small metastasis in the left frontal lobe (arrow) in a 53-year-old female at 3 T, Gadovist 0.1 mL/kg. Postresection changes with an implantation metastasis in the left occipital region (arrowhead). The SE sequence was acquired first

A total of 135 metastatic lesions were identified in all subjects and considered as the true number of lesions (mean per subject 2.41, SD 6.4, range 0–46). Zero metastatic lesions were identified in 22 subjects, while > 1 metastases were detected in 16 subjects. Examples of metastases on SE and SPACES sequences at 1.5 T and 3 T are shown in Figs. 1, 2, 3, and 4. No significant difference in number of metastatic lesions reported at 1.5 T versus 3 T were detected (*p* = 0.79). Relatively fewer metastases were detected on the sequence temporally closer to contrast administration (irrespective of sequence type); however, the difference did not reach statistical significance at the pooled level (*p* = 0.08), nor at the level of individual raters (R1, *p* = 0.16; R2, *p* = 0.19; R3, *p* = 0.5).

**Fig. 5:**
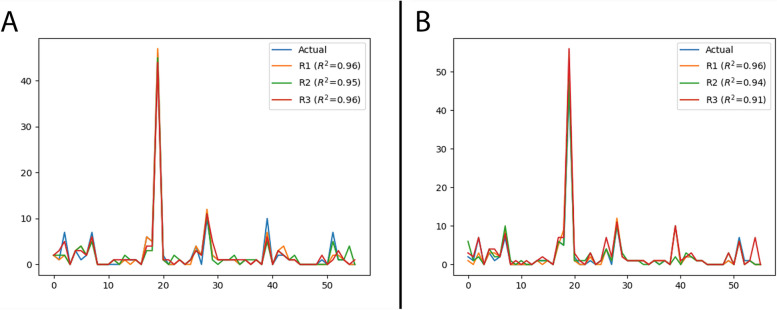
*R*-squared accuracy results for all readers (R1, R2, R3) in comparison to ground truth. **A** T1-weighted SE sequences. **B** T1-weighted SPACE sequences

Intra-rater agreement (SE versus SPACE for each rater) with respect to the number of metastatic lesions detected was excellent in all 3 raters (R1, 0.992 (95% confidence interval (CI) 0.986–0.995); R2, 0.986 (CI 0.975–0.992); R3, 0.97 (CI 0.944–0.983)). Inter-rater agreement (agreement in all raters for SE, agreement in all raters for SPACE) with respect to the number of metastatic lesions detected was also excellent, with ICC values of 0.995 (CI 0.992–0.997) and 0.99 (CI 0.983–0.994) for SE and SPACE sequences, respectively. Inter-rater agreement was poor to moderate for subjective ratings of sequence quality, with SE scoring relatively lower (SE ICC 0.416 (CI 0.009–0.659), SPACE ICC 0.662 (CI 0.37–0.812)); see Table 1. Subjective ratings of sequence quality were also significantly lower for SE, in comparison to SPACE (*p* < 0.001). Accuracy assessment with respect to the number of metastatic lesions detected versus ground truth showed high *r*-squared values for both SE (*R*^2^ 0.95–0.96) and SPACE (*R*^2^ 0.91–0.96) sequences; see Fig. 5 and Table 2. Signal intensity ratios, between enhancing lesions and reference frontal white matter regions, were 2.16 ± 0.66 for SE and 1.75 ± 0.44 for SPACE at 1.5 T and 2.24 ± 0.52 for SE and 1.69 ± 0.31 for SPACE sequences at 3 T; the signal intensity ratios significantly differed at both field strengths (*p* < 0.001).

**Table 1:** Intra- and inter-rater intraclass correlation results

	Intra-rater agreement	Inter-rater agreement			
	*n* metastases	*n* metastases		Quality	
	SE vs. SPC	SE	SPC	SE	SPC
Rater 1	0.992 (CI 0.986–0.995)				
Rater 2	0.986 (CI 0.975–0.992)				
Rater 3	0.97 (CI 0.944–0.983)				
All raters		0.995 (CI 0.992–0.997)	0.99 (CI 0.983–0.994)	0.416 (CI 0.009–0.659)	0.662 (CI 0.37–0.812)

*N* number, *SE* spin echo, *SPC* SPACE, *CI* 95% confidence interval

**Table 2:** Accuracy assessment of ratings by coefficient of determination

	*R*^2^	
	SE	SPC
Rater 1	0.96	0.96
Rater 2	0.95	0.94
Rater 3	0.96	0.91

*SE* spin echo, *SPC* SPACE

## Discussion

The emergence and introduction of fast computer-assisted treatment planning for radiosurgery techniques in the management of brain metastases requires precise, fast, and reliable MR imaging sequences covering the whole brain. Leksell Gamma Knife or CyberKnife, as well as the recently introduced ZAP-X gyroscopic radiosurgery system [17], can treat multiple brain metastases in patients who were previously dependent on whole-brain radiation. However, the fact that a patient underwent contrast-enhanced MRI does not always guarantee the detection of all metastatic lesions, where the choice of imaging sequence is critical.

The SPACE sequence is a single-slab, 3D turbo spin echo (TSE) sequence that uses non-selective, short refocusing pulse trains, allowing for very high turbo factors and sampling efficiency [18]. These T1-weighted 3D acquisitions may therefore be expected to share many advantages with more traditional SE sequences in the detection of metastatic brain lesions, but with shorter acquisition times and greater resolution, while achieving whole-brain coverage and allowing multiplanar reconstruction. Additionally, these sequences are relatively insensitive to susceptibility, flow, and chemical shift artifacts [18].

Published reports regarding the proper use of postcontrast T1-weighted imaging sequences are not consistent. Earlier reports preferred 3D gradient-echo sequences in comparison with thick-slice 2D SE sequences, especially in the detection of small metastatic lesions [13]. This was not due to better quality of enhancement on gradient echo sequences compared to spin echo (the opposite is true [19]), but due to partial volume effects in thick slice acquisitions. Komada et al. [14] compared 3D SPACE, 3D MP-RAGE, and 2D SE sequences with a slice thickness of 4.5 mm and 0.5-mm gap in the imaging of brain metastases at 3 T. They claimed that the results of SPACE and 2D SE sequences were “almost equivalent.” Park et al. [20] found 3D T1 TSE sequences to be superior in comparison with MP-RAGE in the detection of small metastatic lesions at 3 T. Suh et al. [9] provided a meta-analysis of comparative studies examining individual sequences in metastases detection and recommend 3D T1 SE sequence as preferred.

Our results show that the 3D T1-weighted TSE sequence is not inferior to a very thin-slice standard 2D SE sequence in the detection of brain metastases, including very small lesions at the border of detectability. All three experienced readers reached excellent intra- and inter-reader agreement, as well as favorable accuracy in lesion detection on both sequences.

Signal intensity ratios between relatively large (> 4 mm) metastatic lesions and reference white matter were evaluated to quantitatively assess lesion contrast on SE and SPACE sequences. Interestingly, we found that although differences between enhancing lesions and reference white matter on SPACE and SE sequences were statistically significant, with higher contrast between enhancing lesions and reference white matter on SE images, this difference was not clinically relevant. All three blinded readers reached an excellent intra-rater agreement as well as accuracy on both sequences, and inter-rater agreement was also excellent for both sequences. Subjective quality assessment rated SPACE sequences as superior, with relatively better agreement in quality assessment between raters for these sequences compared to SE.

The present study contributes to this topic in several ways. First, to our knowledge, this is the first comparison of a very thin-slice 2D SE (2–3 mm) and 3D T1 TSE sequences in a detailed survey of brain metastases. This is important as 2D SE sequences have been the gold standard for postcontrast imaging of brain metastases for decades. Second, we provide data at two imaging fields, 1.5 T and 3 T. Previous comparative studies were performed at 3 T; the 3D TSE sequence is more challenging to acquire at 1.5 T due to lower signal-to-noise ratio (SNR) [12]. Furthermore, differences not only in SNR, but also in T1 and T2 relaxation times, and different contrast agent relaxivities complicate the comparison of acquisitions at these two field strengths [21]. Acquisitions at 1.5 T are often used in stereotactic targeting due to smaller geometric distortions in comparison with 3 T [22]. Finally, we show that statistically significant differences in lesion contrast (signal intensity ratios) were not clinically relevant.

## Data Availability

All data used in the present study are stored locally at Na Homolce Hospital. Anonymized data may be made available upon reasonable request.

## Abbreviations

GE: Gradient echo
ICC: Interclass correlations
MBL: Metastatic brain lesions
MP-RAGE: Magnetization-prepared rapid gradient echo
ROI: Region of interest
SAR: Specific absorption rate
SE: Spin echo
SNR: Signal-to-noise ratio
SPACE: Sampling perfection with application-optimized contrasts using different flip angle evolution
TSE: Turbo (fast) spin echo
VISTA: Volume isotropic turbo spin echo acquisition
WBR: Whole-brain radiation
